# Loading lime by‐product into derivative cellulose carrier for food enrichment

**DOI:** 10.1002/fsn3.1082

**Published:** 2019-06-04

**Authors:** Rana Afkhami, Mohammad Goli, Javad Keramat

**Affiliations:** ^1^ Department of Food Science and Technology, Isfahan (Khorasgan) Branch Islamic Azad University Isfahan Iran; ^2^ Department of Food Science and Technology Isfahan University of Technology Isfahan Iran

**Keywords:** encapsulation, lime by‐product extract, microparticles, orange juice

## Abstract

The objective here is to enrich orange juice through encapsulated lime by‐product extract (LBE) through freeze‐drying, in order to increase lime by‐product consumption, in addition to increasing nutrition value of orange juice. The properties of both the LBE and microparticles are measured. The total polyphenolic compound (TPC) was measured to be 34.5 ± 0.5 (mg gallic acid/g LBE). The obtained value of encapsulation efficiency (EE) was within the 55%–70% range. The encapsulation method was satisfactory. The particle size is within 10–21 μm range, and differences between all treatments were statistically notable (*p* < 0.05). The lack of melting peaks in the thermal profiles by differential scanning calorimeter (DSC) of microparticles confirmed that hesperidin was well embedded in the polymeric cover. According to the sensory evaluations of orange juice which was enriched with LBE microparticles, the bitter taste was not perceived in some treatments.

## 1 INTRODUCTION

A sizable quantity of by‐product is produced by citrus juice extraction industry, which is mainly consumed as animal feed. This by‐product consists of the peels, seeds, and pulp (Lario et al., 2004). Chemical characterization of citrus extract and its by‐products is essentially unique to orange and lemon and lime; while, lime by‐products due to eminent nutrient content such as polyphenolic flavonoids, carotenoids, ascorbic‐citric acid, and some minerals, that is, Ca^2+^, Fe^2+^, and Mg^2+^, have become the study subject of many studies in this field (Esparza‐Martínez, Miranda‐López, & Guzman‐Maldonado, 2016; Wilmsen et al., 2005). The global lime and lemon production in 2014 were about 13 million tones (FAO, 2016), nearly half of which is turned into wet by‐product. Appearance of a relatively high volume of flavonoids in citrus fruits is due to possible combinations among aglycones with a limited number of monosaccharides and disaccharides (Gattuso, Barreca, Gargiulli, Leuzzi, & Caristi, 2007); identified as flavonoid glycosides (vitamin P) similar hesperidin, rutin, flavones, catechin, and quercetin (Neugart et al., 2015). Hesperidin (hesperetin‐7‐O‐rutinoside) is economical and ample in citrus fruits; furthermore, it has anti‐inflammatory, antioxidant, anti‐carcinogenic, and neuroprotective effects (Bayomy, Elshafey, ElBakary, & Abdelaziz, 2014). Hesperidin is the most abundant and effective component in lime making measurement necessary in lime by‐product extract (LBE). The process of entrapping the active agents is named encapsulation (Nami et al., 2017). The principal purpose of encapsulation is to preserve the core material from inopportune environmental conditions (Mokhtari et al., 2018), for have a long shelf life and supporting a controlled release of the encapsulate (Kim, Lee, & Lee, 2015; Wang et al., 2015). Freeze‐drying is one of the most regularly used encapsulation methods for active compounds, where the core materials homogenize in matrix liquids and then colyophilized, regularly resulting in unpredictable forms. Without for the long dehydration period required (commonly 20 hr), freeze‐drying is a simplistic method for encapsulating water‐soluble extracts, natural aromas, and medicines (Fang & Bhandari, 2010; Nedovic et al., 2011). It is found that at a pH of 2.8–4, cellulose acetate phthalate (CAP) does not dissolve in pH above 7 (gut pH) (Mayhew et al., 2009). The supplemental compounds sodium carboxy methyl cellulose (SCMC), sodium dodecyl benzene sulfonate (SDBS), and xanthan gum are used in order to improve the dissolvability of microparticles in the gut (Lauro et al., 2015). These compounds were chosen from different addictive categories of SCMC, SDBS hydrocolloid xanthan gum, surfactant, and gum, in order to find out which one of the components increase the encapsulation efficiency and yield.

The objective of this project was to identify a by‐product which is rich of hesperidin and flavonoid glycosides and would increase lime by‐product consumption and the orange juice nutrition value. But LBE has a bitter taste and sensitive to a thermal treatment; thus, it should be encapsulated.

## 2 MATERIALS AND METHODS

### 2.1 Materials

The matters consumed include lime by‐product of Rudan city (Persian lime) with harvest time 2016 also CAP, SCMC, xanthan gum, 2,2‐diphenyl‐1‐picrylhydrazyl (DPPH) and hesperidin from Sigma‐Aldrich and Folin–Ciocalteu and gallic acid from Merck and orange juice concentrate (Thompson navel variety) from Chinoud company produced in 2017.

### 2.2 Methods

#### 2.2.1 LBE extraction

The lime by‐product was dried and grounded (Katsampa, Valsamedou, Grigorakis, & Makris, 2015; Ma & Ye, 2008). Sixteen gram of this product was added to 100 ml of ethanol (70%), and the mix was treated with ultrasonic (60 Hz, 200 W, 50°C for 2 hr) and was filtered through Whatman filter papers of 125 mm and treated to the rotary vacuum evaporator at 40°C (IKA RV 10D). Then, it was extracted and freeze‐dried (Dena Tehran, Iran vacuum at 0.98 × 106 bar, and −40°C condenser temperature) (Inoue, Tsubaki, Ogawa, Onishi, & Azuma, 2010; Khan, Abert‐Vian, Fabiano‐Tixier, Dangles, & Chemat, 2010).

#### 2.2.2 LBE physicochemical properties

The pH, color, λ max, and the total phenolic content (TPC) were determined through the process used by Afkhami, Goli, and Keramat (2018). To ensure the efficiency of extraction, the freeze‐dried LBE was proportional to the original lime by‐product weight by percentage. For DPPH assay, the free radical scavenging activity (FRSA) of LBE was assessed through the model presented by (Khan et al., 2010) with some modifications (Afkhami et al., 2018). The total FRSA of each extract was expressed as the percentage of DPPH determined by the following equation:

(1) $$\text{FRSA}=\left[\left(\text{initial absorbance}-\text{final absorbance}\right)/\text{initial absorbance}\right]\times 100$$

The initial and final absorbance constitutes the absorbance values of DPPH at time zero and after 60 min, respectively.

#### 2.2.3 The content of hesperidin

Hesperidin was assessed through high performance liquid chromatographic (HPLC) (series 1100 from Agilent). The HPLC system consists of a (Ultraviolet [UV]) detector, and the separation was applied on a C18 (5 μm‐ 150 × 4.5 mm) column. The HPLC system was equilibrated with the mobile phase consisting of water, methanol, and acetic acid at 40:58:2 ratio, at a 1 ml/min flow rate at 40°C. The LBE without any floating particles was dissolved in distilled water, and then, 20 µl was injected into the HPLC system and the chromatographic peaks were determined at 288 nm. A stock solution of hesperidin was prepared on daily basis by dissolving suitable volumes of the compounds in methanol to achieve final concentrations of 1,000 µg/ml. The spike method was adopted to calculate the hesperidin level; providing the content of hesperidin in LBE to be spiked with suitable volumes of hesperidin stock solution in obtaining the control samples (Afkhami et al., 2018).

#### 2.2.4 Capsule preparation

The CAP was first suspended in 50 ml of distilled water at (2 g) weight next, and 10 mol/L of NaOH was added drop by drop until the CAP dilution was completed, and then, 0.5 g of each SCMC, SDBS, Xanthan gum separately in 50 ml of distilled water containing 0.67 g of dried LBE + 0.027 g of hesperidin was dissolved and then was added to the 50 ml of 4% CAP solution (including 2 g of CAP). So that ratio of LBE to CAP was 1 to 3. The reason why hesperidin was added to LBE is to obtain the hesperidin volume to supplement pills like Geriatric Pharmaton. Each of the formulations was homogenized through Ultra Turrax (IKA T25) at 3,195 g for 15 min in order to yield a stable solution and be freeze‐dried for 24 hr.

#### 2.2.5 Capsule properties

##### Yield of process (Y) and encapsulation efficiency (EE)

To assure the Y, the freeze‐dried solutions were proportional into the initial dry matter weight: the sum of LBE, CAP, and SCMC or SDBS or xanthan gum by percent. For EE, a total of 40 mg of microparticles were added to 2 ml of citrate buffer (0.1 mol/L at pH 3). Then, a total of 40 mg of microparticles were added to 2 ml of phosphate buffer (0.1 mol/L at pH 7.5) to allow the calculation of actual polyphenolic compound. Both the solutions were shaken for 1 min and centrifuged at 4,000 *g* for 10 min in a filtered microtube. To estimate the total initial polyphenolic compound, 100 ml of both the citrate (0.1 mol/L at pH 3) and phosphates buffers (0.1 mol/L, pH = 7.5) was mixed with 0.67 g of dried LBE + 0.027 g of hesperidin. Their polyphenolic compounds were measured as mentioned previously. The EE was calculated through the following equation (Robert et al., 2015; da Rosa et al., 2014; Sansone et al., 2009):

(2) $$\text{EE}=\left[\left(\text{actual polyphenolic compound}-\text{superficial polyphenolic compound}\right)/\text{initial polyphenolic compound}\right]\times 100$$

##### Morphology, particle‐diameter mean analysis, and percentage of water dissolution

The microparticles were coated with gold and platinum and were photographed at varied sizes by the scanning electron microscopy (SEM, Philips × 130) (Lauro et al., 2015). Particle‐diameter mean (μm) and its dispersion (span according to Equation 3) were assessed by Zheng et al. method (2011).

(3) $$\text{Span}=\left[\text{d}\left(\text{V}90\right)-\text{d}\left(\text{V}10\right)\right]/\text{d}\left(\text{V}50\right)$$

Percentage of water dissolution was determined through the method Ahmed, Akter, Lee, and Eun (2010) and then determined through the following equation (Choi, Ryu, Kwak, & Ko, 2010):

(4) $$\text{Percentage of capsule water dissolution}=\left(\text{dried supernatant}/\text{total dried matter}\right)\times 100$$

##### Differential scanning calorimeter (DSC) in microparticles

The DSC was made according to an indium‐calibrated model (SPA 449, NETZCH) through which the thermal behavior on samples of raw materials and microparticles characterized by the highest EE are studied. Each sample was placed in an aluminum pan, which was then sealed and pierced. The samples were exposed to one thermal cycle between 25 and 350°C with a 20 ml/min heating rate and nitrogen gas (Lauro et al., 2015). The DSC curve was drowned in 30 min.

##### Stability of encapsulated hesperidin

This process was run at pH 3 (equal as orange extract) in the pasteurizing heat process (at 80°C in 30 s) and then 60‐day storage time (in days 20, 40, and 60) and finally assessed by HPLC method (Afkhami et al., 2018).

##### Production of enriched orange juice and measurement of sensory characteristics and color

This orange juice was made from (Brix 12) concentrate. The three formulas of microparticles were combined to this orange juice at 200 mg/100 ml (daily dose 20–25 mg), while the fourth formula of LBE (nonmicroparticles) was combined to orange juice at 50 mg/100 ml. The samples were pasteurized at 80°C for the 30 s inside a brown bottle (Polydera et al., 2003) and stored at 4°C. The appearance and taste were evaluated 1 day after production using the seven‐point hedonic test by 15 specialist panelists (Pala & Toklucu, 2013). The color measuring test was run by HunterLab (color meter Zє6000), and the results were taken per L*, a*, and b* factors.

### 2.3 Statistical analysis

The random design was utilized for encapsulation in three replications. To analyze the findings, the SAS 904 software was applied. The differences in mean were defined through Duncan's multiple‐range tests (*p* < 0.05). The data were manifested in the form of mean ± standard deviation.

## 3 RESULTS AND DISCUSSION

### 3.1 LBE physicochemical characteristics

The efficiency of LBE was 18.75%, and the efficiency of extracted lime by‐product was similar to that of (Loizzo et al., 2012) who yield efficiency of extraction within 13%–20%. The TPC of LBE as 34.5 ± 0.5 (mg gallic acid/g LBE) or 646.88 (mg gallic acid/100 g lime [dry basis]) through the standard diagram of gallic acid (*y* = 0.0086*X* + 0.1143 and *R* ^2^: 0.995). TPC content in LBE was like to the volume found in some studies (Esparza‐Martínez et al., 2016; Kuljarachanan, Devahastin, & Chiewchan, 2009) who yield TPC around 15–36 (mg/g [dry weight of LBE]) and 572 (mg/100 g dry basis), respectively. Furthermore, the differences between TPC levels were due to the differences in process conditions. Hesperidin levels in LBE were measured as 2 ± 0.5 (mg/g LBE). It is reported that the content of hesperidin in lime vary in great ranges: as to (Loizzo et al., 2012) who reported hesperidin in Italian limes at very low concentrations (0.05 mg/100 g dry weight) and as to (Saeidi et al., 2011) their hesperidin and eriocitrin in Iranian lime juice was between 10 and 20 µg/ml. While hesperidin levels of (213.87–95.37 [mg/g]) found by Esparza‐Martínez et al., (2016) were more than the ones found in this study, it was revealed that the hesperidin content is more in Mexican lime than Persian lime; furthermore, Peterson et al. (2006) reported hesperidin level of (5–43 [mg/100 g lime]) in different types of lime with a similar result to that of this study. The FRSA was estimated as a 40% reduction in DPPH‐free radical, and the results of (Khan et al., 2010) revealed that the antioxidant activity of orange (*Citrus sinensis* L) peel extract was less than that of the LBE. The λ max, pH, and the LBE color were measured at 291 nm, 3, and L* = 14.46, a* = −0.93, and b* = 5.88 (Brix: 32), respectively.

### 3.2 Freeze‐dried encapsulation: properties and characteristics

**Figure 1:**
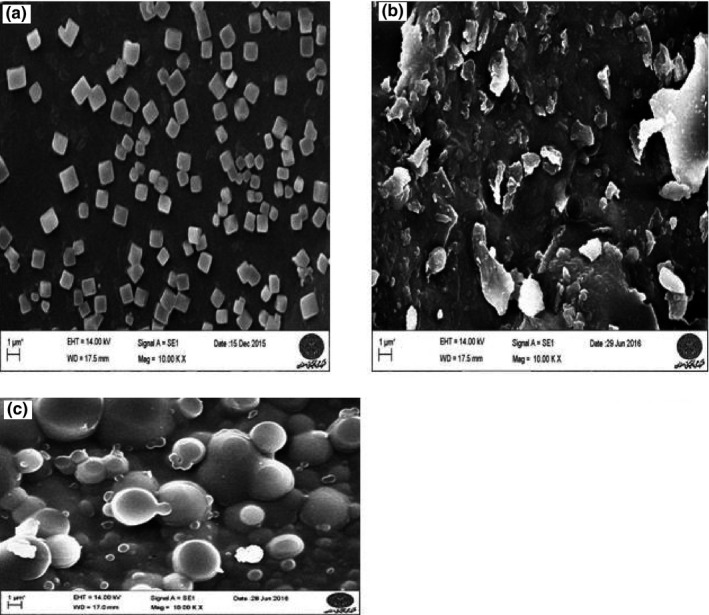
Image from scanning electron microscopy (SEM) of encapsulated polyphenolic extract of lime waste and hesperidin; (a): [CAP/LBE/SCMC], (b): [CAP/LBE/Xanthan], and (c): [CAP/LBE/SDBS]; CAP, cellulose acetate phthalate; LBE, lime by‐product extract; SCMC, sodium carboxy methyl cellulose; SDBS, sodium dodecyl benzene sulfonate

**Figure 2:**
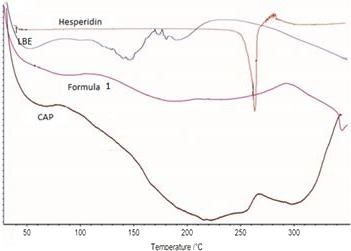
Differential scanning calorimeter (DSC) of Hesperidin, lime by‐product extract (LBE), Formula 1 (SCMC/LBE/CAP), and cellulose acetate phthalate (CAP). SCMC, Sodium carboxy methyl cellulose

Some capsule characteristics were listed in Table 1. The differences between all treatments at Y% were significant with (*p* < 0.05). The highest Y% was achieved by Formula 3 when SDBS was used as a supplemental compound covering agent with the least CAP level and LBE because the mix of these three components yields a solution with low viscosity with least cohesion to the container. Here, the results of yield in my study were similar to the results of Sansone et al. (2009, 2011) who discovered that the highest Y was acquired when SDBS was used as a supplemental compound. The variations between all treatments at EE were statistically significant with *p* < 0.05. The results indicate that LBE is efficiently encapsulated in the CAP/enhancer microsystems. The highest EE was yielded through Formula 1; accordingly, when SCMC was used as a supplemental cover agent, more active compounds were entrapped (Table 1). So that Sansone et al. (2009, 2011) observed that the highest hesperidin and quercetin EE were fixed in microparticles when SCMC was used as a supplemental compound, while the percentages of EE were different because they estimated EE by another equation. The differences between all treatments at particle size were statistically notable at *p* < 0.05, Table 1. The least particle size was observed in Formula 2 and the greatest in Formula 3, indicating that the difference in supplemental coverage material type influence particle size. The LBE might have produced a solid physical mix with SDBS, thus, keeping their crystalline nature. The presence of crystals and aggregates explains the acquired greater sizes. As to particle size, the ones found in this article were alike to that of Scarfato et al. (2008). The observed diversity can be associated with the diversity in encapsulation methods. The lowest span was given by Formulas 1 and 3 (*p* > 0.05), where the particle size was more uniform than the others in terms of its span amount, indicating that homogenizer makes more uniform treatments than others. The results of span here approximately alike to those reported by Sansone et al. (2009, 2011), who discovered that the lowest span when they used SDBS in CAP. As observed in Table 1, the presence of xanthan and SCMC in all formulations leads to a reduction in water dissolution, while the contrast holds true in the presence of SDBS. The SEM results were shown in Figure 1 where the microparticles in Formula 1 were square‐rectangular, multiseeded, and dense, 1A, the microparticles in Formula 2 were shapeless, cohesive, and multiseeded clusters, 1B, and the microparticles in Formula 3 were globular, multiseeded clusters with a smooth surface, 1C. The difference in supplemental coverage, which generated viscosity in solvents, reduced the effect of homogenizer mixer when it comes to in shaping particles. The freeze‐dried method generated irregular and unformed particles in some formulations (Fang & Bhandari, 2010), whereas the spray‐dried method generated shaped particles (Lauro et al., 2015; Sansone et al., 2009, 2011). The DSC results were shown in Figure 2 (Figures S1, S2 and S3), moisture evacuation CREATED due to strong affinity of applied carbohydrates to water generated endothermic peaks at 25–150°C in Formula 1, LBE, and CAP, while depolymerized and pyrolytic decomposition revealed exothermic peaks (between 150−200°C in LBE, 250−300°C in Formula 1, and CAP); hence, depolymerized and pyrolytic decomposition did not occur until 250°C in the microparticles. Hesperidin melted at 260°C (sharp peak in Figure 2), and the absence of these melting peaks in the thermal profiles of microparticles confirms that hesperidin was encapsulated well in the matrices, Figure 2. The results of DSC here were alike to that of published by Lauro et al. (2015) and Sansone et al. (2009, 2011).

**Table 1:** Formulations and encapsulation efficiency% (EE), yield of process% (Y), particle size (µm), span, and water dissolution % of freeze‐dried microparticles

Formula	CAP (g/100 ml)	SCMC (g/100 ml)	Xanthan (g/100 ml)	SDBS (g/100 ml)	LBE (g/100 ml)	EE%	Y%	Particle size	Span	Water dissolution%
1	2	0.5			0.67	70.9 ± 0.1^A^	90.7 ± 0.2^B^	16.25 ± 0.66^B^	1.78 ± 0.2^C^	33.33 ± 1.8^A^
2	2		0.5		0.67	64.3 ± 0.6^B^	88 ± 0.3^C^	10.66 ± 0.57^C^	2.56 ± 0.2^A^	31.25 ± 0^B^
3	2			0.5	0.67	55.1 ± 0.43^C^	91.7 ± 0.2^A^	21.4 ± 0.74^A^	1.83 ± 0.01^BC^	34.37 ± 0^A^

Note

Nonsimilar capital letters in each column indicate significant difference (*p* < 0.05). The results were expressed as the means of three determinations ± standard deviations (*SD*).

Abbreviations: CAP, Cellulose acetate phthalate; LBE, lime by‐product extract; SCMC, sodium carboxy methyl cellulose; SDBS, sodium dodecyl benzene sulfonate.

### 3.3 Evaluating hesperidin stability due to pasteurization heat and 60‐day storage

Percentage of hesperidin damage in control treatments due to heat treatment was 80. Hesperidin damage in Formulas 1, 2, and 3 was around 20, 24, and 34 percent, respectively. Hesperidin was damaged to a larger extent during the pasteurization process if the quantity of surface hesperidin would have increased. During 60‐day storage, the hesperidin was not damaged because hesperidin can undergo refrigeration condition (4°C) without any notable change. The previous results of hesperidin stability were not shown any significant damage in hesperidin during storage; as result, they conclude that hesperidin is resistant to storage conditions (Fathi & Varshosaz, 2013; Lauro et al., 2015; Sansone et al., 2009).

### 3.4 Evaluating the sensory properties and color of the enriched orange juice

**Figure 3:**
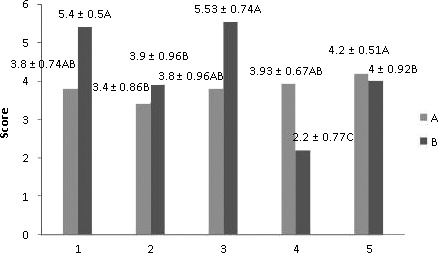
The sensory assessment of treatments in the first day after production in orange juice, Treatment 1: ([CAP/LBE/SCMC] + orange juice), 2: ([CAP/LBE/Xanthan] + orange juice), 3: ([CAP/LBE/SDBS] + orange juice), 4: [LBE + orange juice], and 5: [Control = orange juice] (*n* = 15, similar capital letters indicate lack of significant difference [*p* < 0.05]), CAP, cellulose acetate phthalate; LBE, lime by‐product extract; SCMC, sodium carboxy methyl cellulose; SDBS, sodium dodecyl benzene sulfonate; A: taste and B: appearance feature

The results of the sensory evaluation of treatments on the first day after production in orange juice were shown in Figure 3. The lowest score was obtained in Treatment 2: ([CAP/LBE/Xanthan] + orange juice), while other scores of encapsulated treatments were similar to the control; the difference between Treatment 2 and the control was statistically notable at *p* < 0.05. The highest score in the taste was obtained in Treatments 1: ([CAP/LBE/SCMC] + orange juice) and 2: ([CAP/LBE/SDBS] + orange juice), better than the control, indicating that, encapsulation removed the bitter taste. The results of this research were alike to that of Fathi and Varshosaz (2013) where milk was fortified with hesperetin‐loaded nano‐carriers, and they did not obtain any notable differences with the control. The results of the color parameters (L*, a*, and b*) in all freeze‐dried microparticles formula in 60‐day storage are tabulated in Table 2. The L* factor that shows color lightness was increased in all treatments in a meaningful manner when compared with the control, as shown in Table 2; the L* factor in Treatments 3: ([CAP/LBE/SDBS] + orange juice) and 4: [LBE + orange juice] was more than other treatments at days 0, 20, 40, and 60. The a * factor that reflects green–red in all treatments was not significant at *p* < 0.05 when compared with control, Table 2. The b* factor that indicates blue–yellow was significantly (*p* < 0.05) enhanced in all treatments when compared with control; furthermore, b* factor in Treatments 3: ([CAP/LBE/SDBS] + orange juice) was more than other treatments at days 0, 20, 40, and 60. The a * and b* factors in most treatments follow the equal pattern when compared with the control sample at 60‐day storage. The results of this study were alike to that of (Cortes, Esteve, & Frígola, 2008).

**Table 2:** The color parameters (L*, a*, and b*) in all freeze‐dried microparticles formula in 60‐day storage

	Storage time (day)											
	0			20			40			60		
	L*	a*	b*	L*	a*	b*	L*	a*	b*	L*	a*	b*
1	26.4 ± 0.33^h^	−1.01 ± 0.43^efgh^	15.89 ± 0.27^ef^	26.61 ± 0.48^gh^	−0.6 ± 0.13^abcde^	15.18 ± 0.43^g^	27.76 ± 0.25^f^	−1.2 ± 0.1^fgh^	15.76 ± 0.11^f^	27.70 ± 0.26^f^	−0.65 ± 0.18^abcde^	14.81 ± 0.13^gh^
2	25.61 ± 0.21^i^	−0.63 ± 0.55^abcde^	14.06 ± 0.78^i^	25.25 ± 0.16^i^	−0.82 ± 0.03^bcdef^	14.27 ± 0.55^hi^	27.02 ± 0.08^g^	−0.93 ± 0.06^cdefg^	17.11 ± 0.24^bc^	23.95 ± 0.12^j^	−0.43 ± 0.2^ab^	14.22 ± 0.21^i^
3	29.76 ± 0.1^ab^	−0.76 ± 0.13^bcdef^	17.37 ± 0.46^b^	30.04 ± 0.22^a^	−0.49 ± 0.05^abc^	16.98 ± 0.31^bc^	30.17 ± 0.28^a^	−1.04 ± 0.06^efgh^	18.69 ± 0.27^a^	29.35 ± 0.07^bc^	−0.51 ± 0.19^abc^	16.77 ± 0.29^cd^
4	28.45 ± 0.47^e^	−0.76 ± 0.4^bcdef^	16.24 ± 0.06^def^	28.3 ± 0.58^e^	−0.83 ± 0.42^bcdef^	16.34 ± 0.06^de^	29.11 ± 0.23^cd^	−1.03 ± 0.06^defgh^	17.2 ± 0.14^bc^	28.73 ± 0.09^de^	−0.57 ± 0.16^abcd^	15.84 ± 0.26^ef^
5	21.55 ± 0.35^l^	−0.99 ± 0.18^defgh^	12.13 ± 0.59^k^	22.12 ± 0.3^k^	−0.81 ± 0.17^bcdef^	11.71 ± 0.15^kl^	22.39 ± 0.14^k^	−1.28 ± 0.39^gh^	13.06 ± 0.17^j^	22.07 ± 0.08^k^	−0.59 ± 0.16^abcde^	11.53 ± 0.29^l^

Note

Nonsimilar letters in each factor (L*, a*, and b*) during 60‐day storage indicate significant difference (*p* < 0.05). The results were expressed as the means of three determinations ± standard deviations (*SD*). Treatment 1: ([CAP/LBE/SCMC] + orange juice], 2: ([CAP/LBE/Xanthan] + orange juice), 3: ([CAP/LBE/SDBS] + orange juice), 4: [LBE + orange juice], and 5: [Control = orange juice].

Abbreviations: CAP, cellulose acetate phthalate; LBE, lime waste extract; SCMC, sodium carboxy methyl cellulose; SDBS, sodium dodecyl benzene sulfonate.

## 4 CONCLUSION

The obtained results here indicate that LBE entrapment in microparticles is within 55%–70%, thus, successful encapsulation. Consuming different supplemental agents affect the EE and when SCMC was consumed as an auxiliary covering agent, more active compounds were entrapped. According to the sensory characteristics of orange juice, enriched with LBE microparticles, the bitter taste was removed in some treatments. Hence, this encapsulation method is contributive in removing the bitter taste. Future proposals of this study consist of running new studies on other encapsulation methods like extrusion, encapsulation of other fruit waste extracts by this method, a clinical study run on the effect of this enriched orange juice on the human.

## CONFLICT OF INTEREST

The authors declare that they do not have any conflict of interest.

## ETHICS STATEMENT

This study does not involve any human or animal testing.
