# Supplementary figures and images for: Loading lime by‐product into derivative cellulose carrier for food enrichment
Source: Food Sci Nutr. 2019 Jun 4;7(7):2353–60. doi: 10.1002/fsn3.1082 (PMC6657750; doi:10.1002/fsn3.1082)

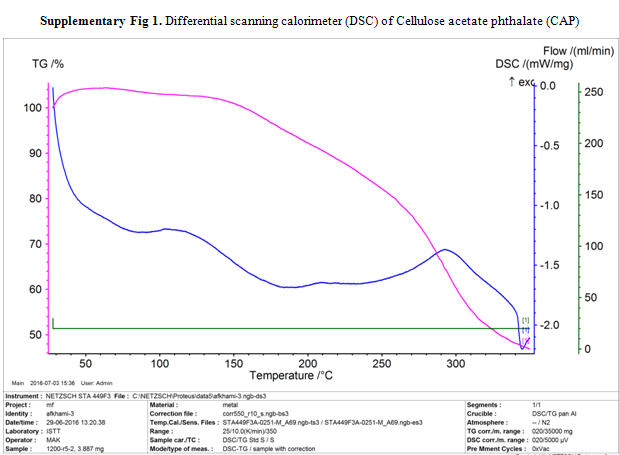

Supplement: Supplementary file 1 [file FSN3-7-2353-s001.TIF]

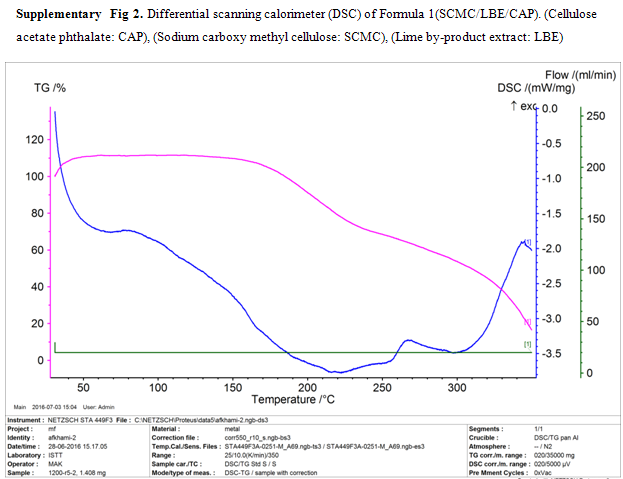

Supplement: Supplementary file 2 [file FSN3-7-2353-s002.TIF]

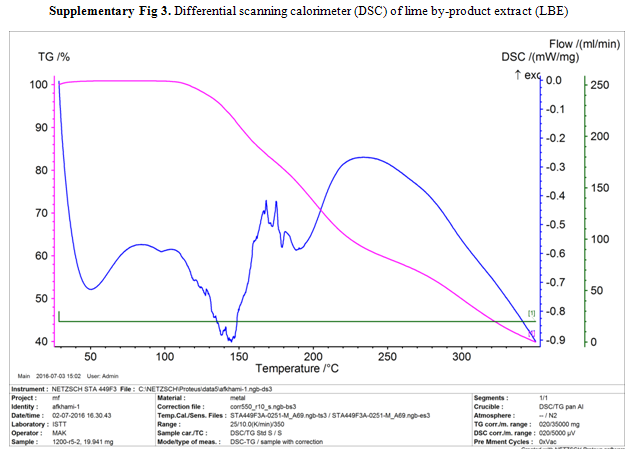

Supplement: Supplementary file 3 [file FSN3-7-2353-s003.TIF]
